# Biocompatibility of Polypyrrole with Human Primary Osteoblasts and the Effect of Dopants

**DOI:** 10.1371/journal.pone.0134023

**Published:** 2015-07-30

**Authors:** Anna Fahlgren, Cornelia Bratengeier, Amy Gelmi, Cornelis M. Semeins, Jenneke Klein-Nulend, Edwin W. H. Jager, Astrid D. Bakker

**Affiliations:** 1 Department of Clinical and Experimental Medicine, Division of Orthopaedics, Linköping University, Linköping, Sweden; 2 Department of Physics, Chemistry and Biology, Biosensors and Bioelectronics Centre, Linköping University, Linköping, Sweden; 3 Department of Oral Cell Biology, ACTA-University of Amsterdam and VU University Amsterdam, MOVE Research Institute Amsterdam, Amsterdam, The Netherlands; Second University of Naples, ITALY

## Abstract

Polypyrrole (PPy) is a conducting polymer that enables controlled drug release upon electrical stimulation. We characterized the biocompatibility of PPy with human primary osteoblasts, and the effect of dopants. We investigated the biocompatibility of PPy comprising various dopants, i.e. *p*-toluene sulfonate (PPy-pTS), chondroitin sulfate (PPy-CS), or dodecylbenzenesulfonate (PPy-DBS), with human primary osteoblasts. PPy-DBS showed the roughest appearance of all surfaces tested, and its wettability was similar to the gold-coated control. The average number of attached cells was 45% higher on PPy-DBS than on PPy-CS or PPy-pTS, although gene expression of the proliferation marker Ki-67 was similar in osteoblasts on all surfaces tested. Osteoblasts seeded on PPy-DBS or gold showed similar vinculin attachment points, vinculin area per cell area, actin filament structure, and Feret’s diameter, while cells seeded on PPY-CS or PPY-pTS showed disturbed focal adhesions and were enlarged with disorganized actin filaments. Osteoblasts grown on PPy-DBS or gold showed enhanced alkaline phosphatase activity and osteocalcin gene expression, but reduced osteopontin gene expression compared to cells grown on PPy-pTS and PPy-CS. In conclusion, PPy doped with DBS showed excellent biocompatibility, which resulted in maintaining focal adhesions, cell morphology, cell number, alkaline phosphatase activity, and osteocalcin gene expression. Taken together, conducting polymers doped with DBS are well tolerated by osteoblasts. Our results could provide a basis for the development of novel orthopedic or dental implants with controlled release of antibiotics and pharmaceutics that fight infections or focally enhance bone formation in a tightly controlled manner.

## Introduction

Although the current initial success rate of dental implants is high, there are complications associated with dental implant placement, e.g. peri-implantitis, which results in tissue degeneration close to the implants due to inflammatory processes. The incidence of peri-implantitis varies between 7 and 17% depending on the implant type and bone quality five to ten years after surgery [1]. Considering the high number of implants placed within the last decade, the absolute number of expected new cases of peri-implantitis is extremely high. Peri-implantitis causes extensive and costly revisions, and no preventive measures currently exist. The survival rate of implants is even lower when placed for the second or third time due to implant loosening, and therefore clinicians tend to avoid dental implant placement peri-implantitis cases. Thus, a controlled release of antibiotics and/or growth factors from the implant surface over an extended time after implantation could provide a promising treatment option. Currently, many devices are under development for controlled drug release. Most of these devices are limited with respect to the amount of control that can be exerted over the timing and kinetics of the release. Conjugated or conducting polymers are promising materials due to their high biocompatibility and electrical conductivity. The surface topography, wettability, and mechanical properties can be altered depending on the type of dopants and the synthesis method [2–4]. Using a conducting polymer with biological active molecules or drugs in combination with electric stimulation would open up treatment strategies for controlled drug release from bone implants [5]. The conducting polymer polypyrrole (PPy) doped with bioactive agents supports the viability of tissues and cells including osteoblasts [6], cardiac progenitor cells [7], endothelial cells, renal epithelial cells [8], keratinocytes, fibroblasts, neurons, and mesenchymal stem cells [9]. However, only limited knowledge is available on how the osteogenic potential and activity of human primary osteoblasts is influenced by different dopants. In the current study, the biocompatibility of the conducting polymer PPy synthesized with three different dopants on human osteoblasts was determined. During electrosynthesis the conducting polymer is oxidized, and a negatively charged counter ion from the electrolyte solution is incorporated to balance the charge [10]. Due to its analogy with the doping of other semiconductors such as silicon, this counter ion is often addressed as a dopant. The biologically active molecule chondroitin sulfate (CS) was chosen since it plays a key role in the attachment and adhesion of osteoblasts to the extracellular matrix (ECM) in bone [11]. CS is actively produced by osteoblasts and stored in their membrane, thereby preventing osteoblast-mediated activation of osteoclasts [12]. Dodecylbenzenesulfonate (DBS) and p-toluene sulfonate (pTS) are simple aromatic dopants that are commonly used to synthesize PPy with good material properties, such as high electrical conductivity and stability [13–15]. In addition, these dopants have been extensively characterized during the last years, using a broad variation of different cell types, thereby providing good controls for comparison. Our primary goal was to find a biocompatible surface for bone implants, and therefore we compared the biocompatibility of PPy incorporated with CS, DBS, or pTS in relation to a gold control surface, in primary osteoblast cultures. The primary osteoblasts were harvested from three patients who underwent primary hip arthroplasty. The overall aim was to find a dopant that is biocompatible with human osteoblasts, on which the cells remain viable and keep their osteogenic function, and that has the potential to be used as a drug delivery system. We determined how these dopants affect osteoblasts in terms of cell attachment and morphology, viability, proliferation, and osteogenic stimulation potential to provide fundamental knowledge for optimization of bone implant surfaces for osteogenic cells and ultimately bone (re)generation.

## Material and Methods

### Synthesis of biomaterials

Si wafer pieces were coated with a 100 nm Au and a 5 nm Ti adhesion layer using thermal evaporation. The gold-coated Si pieces were cleaned with ethanol and subsequently rinsed with ultrapure RNAase-free water (18 MΩ). PPy was synthesized on 1 or 4 cm^2^ of the Au pieces using electrochemical polymerization in a three electrode electrochemical cell using a stainless steel mesh counter electrode and an Ag/AgCl reference electrode (BASi, West Lafayette, IN, USA). Since only part of the Au sample was covered with the conducting polymer, we could use the non-coated Au as an integrated control. For all polymerization processes the aqueous monomer solution contained 0.1 M PPy and 2 mg/ml of the appropriate dopant, which were electrochemically synthesized with a current density of 0.5 mA/cm^2^ for 10 min using a Compactstat.e (Ivium, The Netherlands). The dopants used were chondroitin sulfate A sodium salt (Sigma-Aldrich, MO, USA), dodecylbenzenesulfonic acid sodium salt (TCI, Europe), and sodium para-toluenesulfonate (Sigma-Aldrich). After polymerization, the samples were cleaned with ultrapure RNAse-free water (18 MΩ) and dried with N_2_.

### Contact angle goniometry

A CAM200 optical contact angle meter (KSV Instruments, Finland) was used for comparative evaluation of the surface energy of PPy. The sessile drop method using ultrapure RNAase-free water (18 MΩ) was used to measure the interface angle. For each film, three samples were analyzed by measuring the left and right angle of the drop ten times.

### Profilometry

The roughness and thickness of the polymer films were measured using a Dektak 6M profilometer (Veeco Instruments Inc., New York, NY, USA). The roughness was investigated by measuring the surface three times per sample at different locations over a length of 5000 μm with a stylus force of 3 mg. The thickness was measured three times per sample at different locations at the Au PPy boundary.

### Scanning electron microscopy

Scanning electron microscopy (SEM) micrographs were taken using a Leo 1550 Gemini scanning electron microscope operating at 5.02 keV. The samples were coated with a gold layer via evaporation.

### Cell culture

Human primary osteoblasts were harvested from patients undergoing primary hip arthroplasty as described previously [16]. Cells were obtained in an anonymized fashion from surgical waste material, which was forwarded exclusively by the attending physician. According to the Dutch law, neither approval of an ethics committee nor written consent of the patients is required when using surgical waste material. However, the patients were informed and gave verbal consent, which was not further recorded. The protocol for using cells from waste material in the experiments was approved by the Ethical Review Board of the VU University Medical Center, Amsterdam, The Netherlands. Frozen cells of 3 donors at passage 0 or 1 were pre-cultured for 5 days in Dulbecco’s Minimal Essential Medium (DMEM; Life Technology, Orange, CA, USA) with 1% penicillin/streptomycin/fungizone (PSF, Sigma Aldrich), 0.2% heparin 5000 UI/ml (LEO Pharma, Ballerup, Denmark), and 5% platelet lysate (VU University Medical Center, Amsterdam, The Netherlands) at 37°C with 5% CO_2_ in a humidified atmosphere. Osteoblasts were seeded at a density of 5,000 cells/cm^2^ onto the biomaterial samples in DMEM with 1% PSF and 10% FC1 (HyClone, Thermo Scientific, Logan, UT, USA). Cells were allowed to attach and grow until subconfluency for 3 days, at which point 10^−8^ M 1α,25-dihydroxyvitamin D_3_ (1α,25-(OH)_2_D_3_;_;_ Sigma Aldrich) was added to the medium to induce osteogenic differentiation (i.e. start of the experiment, day 0). 1α,25-(OH)_2_D_3_ was only added during the first 3 days of the experiment. Analysis of DNA content, gene expression, and alkaline phosphatase (ALP) activity was performed in duplicate on 4 cm^2^ PPy dopant samples, while analysis of F-actin staining was done in triplicate on 1 cm^2^ polymer samples. Three separate experiments were performed with different donors of human primary osteoblasts.

### F-actin filament staining and analysis of cell morphology

Osteoblasts grown on polymer samples were fixed in 4% formaldehyde at room temperature (RT), and stained using 5 units/ml fluorescent phallotoxin (Alexa Fluor 488 phalloidin (Life Technologies)) in PBS for 30 min. The stained samples were washed twice with PBS, and stored at 4°C. Changes in cell morphology were detected by fluorescence microscopy. Images were taken using Leica DMRA fluorescence microscope (Leica Microsystems, Wetzlar, Germany) and Leica QWin Image Software (Leica Microsystems) at a magnification of 20-fold, and used for F-actin image analysis. Cell area, perimeter, and Feret’s diameter were analyzed using Fiji ImageJ (National Institute of Mental Health, MD, USA) [17], after applying the Auto Threshold Triangle Method [18], followed by binarization of the images. Cells that appeared clearly separated were used for further analysis. The number of cells was manually counted in 3 pictures per sample, and was presented as number of osteoblasts/100 μm^2^. The length and number of F-actin filaments were analyzed by using FilaQuant [19], that was kindly provided by Dr. Konrad Engel (University Rostock, Institute for Mathematics, Rostock, Germany). Pictures were pre-processed using Fiji ImageJ (see detailed description provided in S1 File). Subsequently, the color channels were split, and the result of the green channel was used for further analysis by FilaQuant. To avoid skewing of the data due to fragmented F-actin filaments, a minimum length of 2.5 μm was taken for statistical analysis. The settings for analyzing the F-actin filaments are provided in S1 Table.

### Vinculin staining and analysis of focal adhesions

After fixation with 0.4% formaldehyde of osteoblasts grown on polymer samples, vinculin was detected by monoclonal anti-vinculin produced in mouse clone hVIN-1 (Sigma) at a 1:400 dilution for 1h at RT in PBS with 0.5% BSA (Sigma) and 0.025% Triton-X100 (Merck). Goat anti-Mouse IgG (H+L) polyclonal antibody conjugated with Alexa Fluor 488 (Life Technologies) at a concentration of 2 μg/ml was used as secondary antibody, and actin filaments were stained with 450 mU/ml fluorescent phallotoxin (Alexa Fluor 568 phalloidin (Invitrogen, Molecular Probes, Eugene, OR, USA)) in PBS with 0.025% Triton-X100 (Merck) for 1 h, dark at RT. Epifluorescence images were taken using an Axiovert 200M inverted microscope (Carl Zeiss, Oberkochen, Germany) with an alpha Plan-Apochromat 63x/1,46 Oil Korr M27 objective, AxioCam MR camera (Carl Zeiss) and ZEN2 Software (Carl Zeiss). The number of vinculin attachment points/100 μm^2^ cell, and the percentage vinculin area/cell area were analyzed using Fiji ImageJ [17] (National Institute of Mental Health, Bethesda, MD, USA) particle analysis feature after applying the Auto Threshold Triangle Method [18] followed by binarization of the images. Only cells that appeared clearly separated were used for analysis. Pictures were pre-processed using Fiji Image J (see a detailed description in S2 File).

### DNA Quantification

The DNA content was measured after 24 h using CyQUANT Cell Proliferation Assay Kit (Life Technologies, Orange, CA, USA) according to the manufacturer’s instructions.

### Alkaline phosphatase (ALP) assay

ALP activity was quantified after 24 h by a p-n-phenyl phosphate colometric assay (Merck, Darmstadt, Germany). The absorbance at 405 nm was measured by a microplate reader. ALP activity values were normalized for DNA content of the respective donor.

### RNA isolation and real-time qPCR

Total RNA was extracted using the TRIzol method. cDNA was prepared using a Revert Aid First Strand cDNA Synthesis Kit (Fermentas, Thermo Scientific, Waltham, MA, USA) according to the manufacturer’s instructions. Gene expression of for the cell proliferation marker KI-67, the bone-specific genes runt-related transcription factor 2 (RUNX2), alkaline phosphatase (ALP), osteocalcin (OCN), Osteopontin (OPN), Osteonectin (SPARC) and Collagen, Type I, Alpha 1 (COL1A), as well as the house-keeping genes hypoxanthine phosphoribosyltransferase 1 (HPRT1) and tyrosine 3-monooxygenase/tryptophan 5-monooxygenase activation protein zeta (YWHAZ) were quantified (Table 1) in a Light Cycler 480 (Roche Diagnostics; Indianapolis, IN, USA). The relative concentration of the gene of interest compared to the HPRT1 and YWHAZ concentration was calculated using a standard curve of serially diluted cDNA to correct for PCR efficiency. The measurement of the gene expression was normalized based on the square root of the product formed by HPRT1 and YWHAZ.

**Table 1:** Primer sequences used for qPCR.

Gene name	Primer sequences 5’–3’	Product size [bp]
**HPRT1**	Fw: GCTGACCTGCTGGATTACAT	260
	Rev: CTTGCGACCTTGACCATCT	
**WHYAZ**	Fw: GATGAAGCCATTGCTGAACTTG	229
	Rev: CTATTTGTGGGACAGCATGGA	
**RUNX2**	Fw: ATGCTTCATTCGCCTCAC	156
	Rev: ACTGCTTGCAGCCTTAAAT	
**ALP**	Fw: GCTTCAAACCGAGATACAAGCA	102
	Rev: GCTCGAAGAGACCCAATAGGTAGT	
**OCN**	Fw: GCCCAGCGGTGCAGAGT	106
	Rev: GGCTCCCAGCCATTGATACA	
**OPN**	Fw: TTCCAAGTAAGTCCAACGAAAG	181
	Rev: GTGACCAGTTCATCAGATTCAT	
**SPARC**	Fw: CTGTCCAGGTGGAAGTAGG	233
	Rev: GTGGCAGGAAGAGTCGAAG	
**COL1A**	Fw: TCCGGCTCCTGCTCCTCTTA	336
	Rev: GGCCAGTGTCTCCCTTG	
**KI-67**	Fw: CCCTCAGCAAGCCTGAGAA	202
	Rev: AGAGGCGTATTAGGAGGCAAG	

### Statistics

Statistical analysis of biomaterial surface characteristics was performed using one-way ANOVA, followed by t-tests with Bonferroni correction for post-hoc comparisons. For experiments with primary human osteoblasts, one-way repeated measured analysis of variance (ANOVA) was performed with paired-samples t-tests for post-hoc comparisons, while data obtained with cells from the same human donor were considered paired. Gene expression and ALP activity data were normalized to values obtained with cells seeded on gold surface of each donor. DNA content and ALP activity values obtained with osteoblasts grown on PPy-CS or PPy-pTS were below the detection limit in cells from 2 out of 3 donors. For statistical analysis, representative data were calculated by using half of the lower limit of the detection of the assay performed [20]. All data are presented as mean ± SD. A p-value <0.05 was considered to be statistically significant.

## Results and Discussion

### Surface characterization

**Fig 1:**
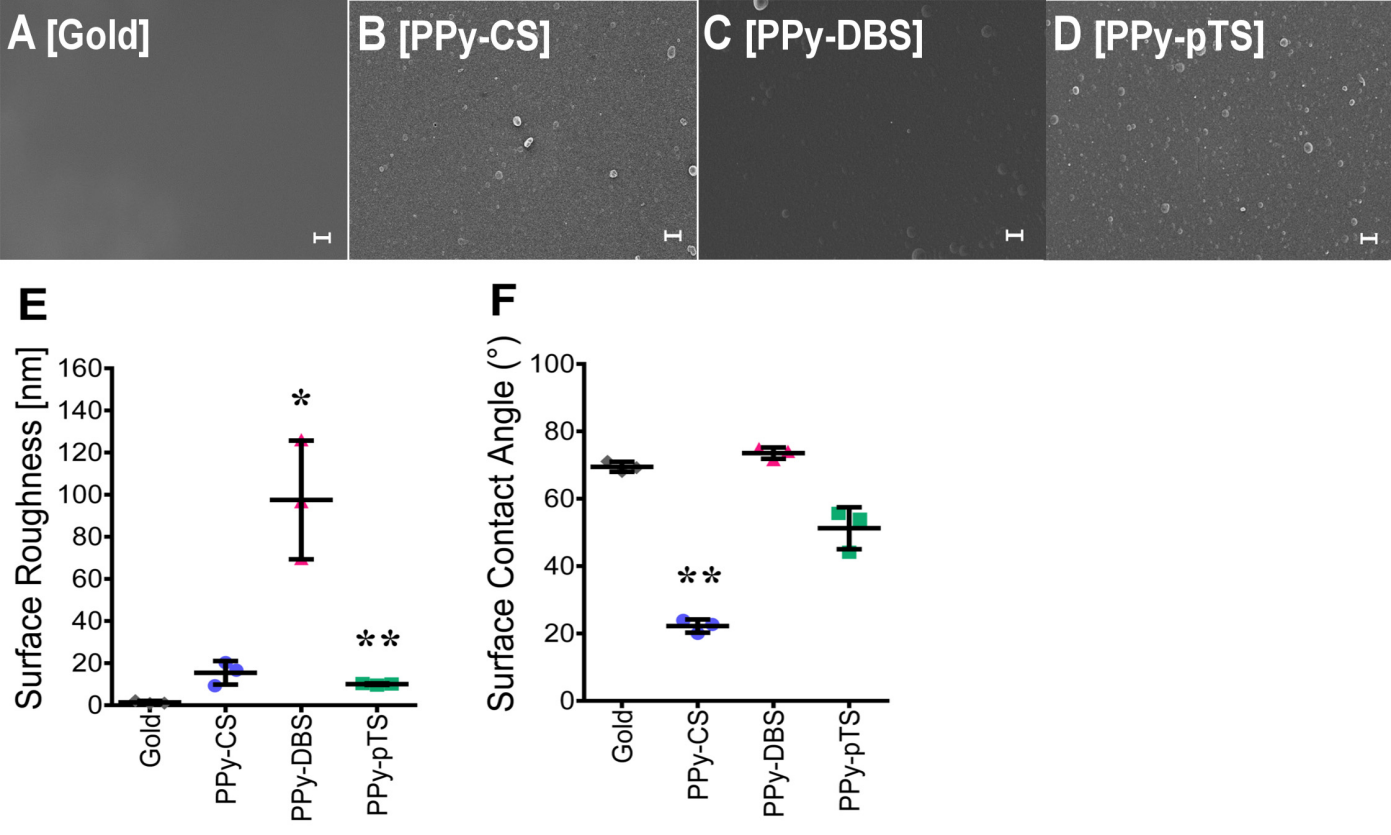
Surface characterization. SEM images of gold [A] and Polypyrrole (PPy) doped with chondroitin sulfate (CS) [B], dodecylbenzenesulfonate (DBS) [C] and p-Toluene sulfonate (pTS) [D]. Contact angle test [E] and roughness analysis [F] of the different surfaces. The value represents mean ± standard deviation (n = 3). **p<0.01, v. gold control. The scale bars represent 2 μm.

Wettability and roughness are important parameters commonly discussed for surface characterization. The choice of dopant has a major influence on the surface roughness and hydrophilicity of electrochemically polymerized polymers [7]. These surface properties in turn affect cell attachment, proliferation, and differentiation potential [21]. The surface properties are not only affected by the structure of the dopant, but also synthesis parameters during electrochemical polymerization play a key role [22]. A direct chemical influence by dopants present within the material slightly affects the ability of cells to adhere to the surface [4], while physical material surface properties have a much greater influence on cellular behavior [2,5]. Surface roughness is important for cell adhesion. Experimental synthesis parameters, including current density (charge passed per area per time), synthesis time, choice of dopant, as well as the dopant and monomer concentration directly affect the roughness and thickness of the polymer [7,13]. In the current study, the roughness and thickness of the polymers were measured by profilometry. The thickness of pTS (250 ± 50 nm) appeared to be thinner than that of CS (560 ± 150 nm) or DBS (620 ± 50 nm). However, these thickness values did not correlate to the roughness of the materials. The roughness value of the polymer Ppy-DBS was different in magnitude (R_a_ = 98 ± 15 nm) (Fig 1E) than that of the other materials tested. pTS (R_a_ = 15 ± 3 nm) was also significantly more rough than gold, but not to the same magnitude as DBS. The gold control surface was smooth (2.2 ± 0.03 nm) without features, as expected for an evaporation-coated silicon surface. However, the roughness values were relatively low for all materials (< 100 nm). This low degree of roughness of a material surface is more preferable for bone cells than flat or micro-scale roughness surfaces [23]. Surface roughness has a significant influence on osseointegration, where nano- and micro-scale structures result in improved osseointegration compared to a smooth surface [24]. Furthermore, an increased roughness value directly influences osteoblast adhesion and proliferation [24]. Osteoblasts attach to a rough surface at more positions with less strength compared to a smooth surface [25]. All of the materials tested demonstrate hydrophilicity. However, the water contact angle values showed that PPy-CS was most hydrophilic of all dopants (22.2 ± 0.2°) compared to gold (68.1 ± 0.2°), whereas hydrophilicity of PPy-DBS (73.5 ± 0.2°) and PPy-pTS (51.2 ± 0.7°) was similar to that of gold (Fig 1F). All materials tested revealed contact angles between 10° and 80°, which is considered normal hydrophilic with good wettability. Hydrophilic surfaces are favorable for anchorage-dependent cells, like osteoblasts, since these result in higher cell proliferation and reduced cell apoptosis rates [26]. Surface topography is also important for cell adhesion and proliferation. SEM images (Fig 1A–1D) showed the very smooth surface and the typical nodular features of PPy. PPy-CS displayed a low density of sub-micron diameter nodules, unlike PPy-pTS, that revealed similar sized nodular features but with a higher nodule density. The diameter of the nodular features on PPy-DBS was approximately 1–2 μm. The control surface of sputter evaporation-coated gold was featureless, as expected. Implant surface microtopography is more important than surface chemistry for early healing and bone regeneration due to the attraction of platelets, which release chemotactic growth factors leading to osteoblast recruitment and bone matrix formation [27]. Variation of physiochemical properties might also influence attachment, viability, and osteogenic properties of osteoblasts [28].

### Cell viability and proliferation

**Fig 2:**
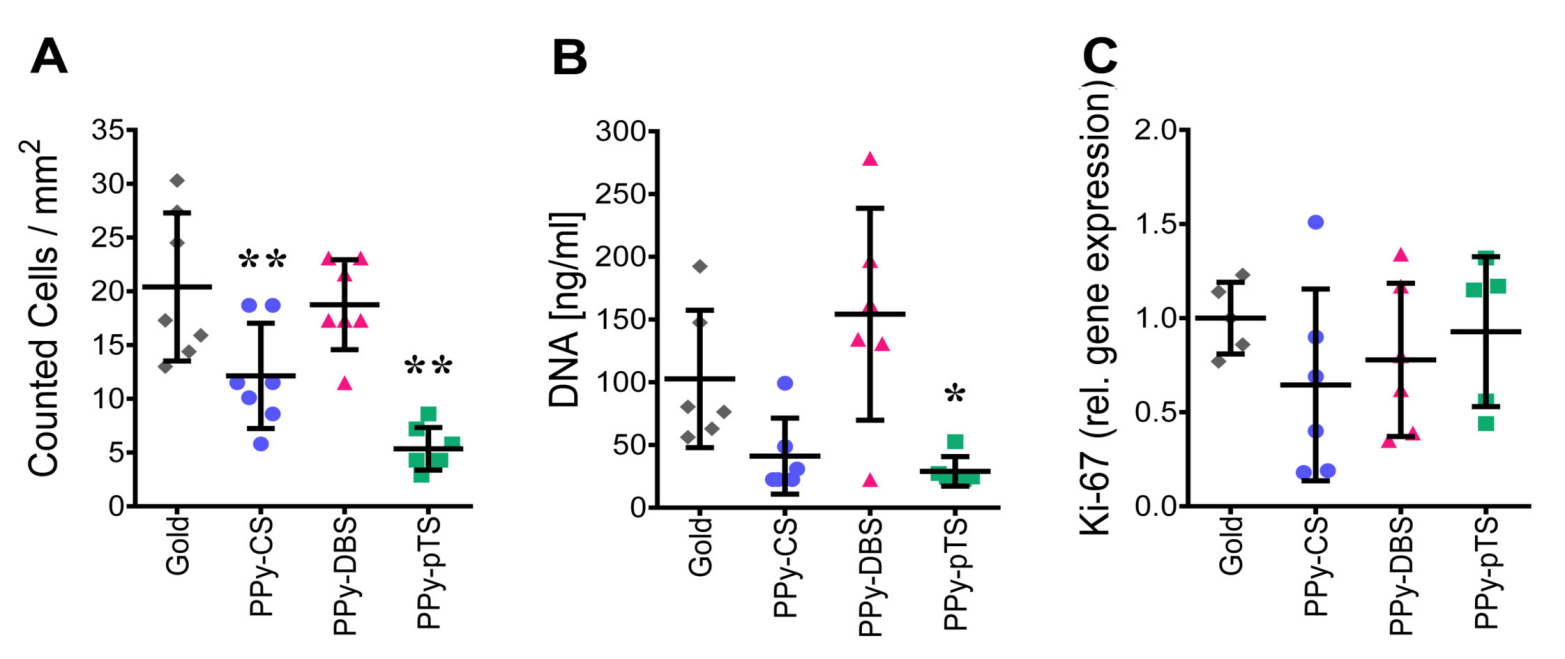
Cell viability and survival. Analysis of cell number/mm^2^ on 1 cm^2^ samples [A], DNA concentration on 4 cm^2^ samples [B] and of the gene expression of the proliferation factor KI-67[C] of gold and Polypyrrole (PPy) doped with chondroitin sulfate (CS), dodecylbenzenesulfonate (DBS) and p-Toluene sulfonate (pTS). The value represents mean ± standard deviation. The value of KI-67 was normalized based on the square root of the product formed by HPRT1 and YWHAZ gene expression and represent mean ± standard deviation normalized to gold control (n = 5). *p<0.05, **p<0.01v. gold control.

Cell viability and proliferation are important parameters in biocompatibility studies. Mostly only partial cell viability parameters are studied, such as life-death staining. Here, we report a detailed analysis of osteoblast viability using DNA content, visualization of cell number per area, and mRNA expression of a proliferation marker to determine potential inhibitory effects of biomaterials [29]. We found that the DNA content and cell number were similar on PPy-DBS and on gold, while both cell number and DNA content were decreased on PPy-CS and PPy-pTS (Fig 2A–2B). The DNA content of osteoblasts attached to PPy-CS and PPy-pTS was below the detection limit in 2 out of 3 donors, indicating a poor adhesion of human osteoblasts to these surfaces. Thus, cells that were poorly attached to the material surface could be easily removed by performing washing steps. Besides the number of primary human osteoblasts attached to the biomaterials, we also determined the proliferative activity of the cells attached to the material surface by analyzing gene expression of KI-67. We did not detect differences in the expression of this proliferation marker KI-67 in cells attached to the different materials (Fig 2C). Therefore we suggest that none of the materials tested did induce cell proliferation. Nevertheless, excellent attachment of bone cells is essential to provide a favorable situation to improve cell proliferation by electric stimulation of the conducting polymer [30].

### Cell attachment and morphology

F-actin and vinculin protein expression were used to investigate the arrangement of the actin cytoskeleton and focal adhesions. In all fields of tissue engineering, cell adhesion and attachment are equally important as are soluble growth factors, cell-cell interaction, and mechanical stimulation [31]. Cells that attach properly do form a healthy environment that supports osteoblast activity and osseointegration.

**Fig 3:**
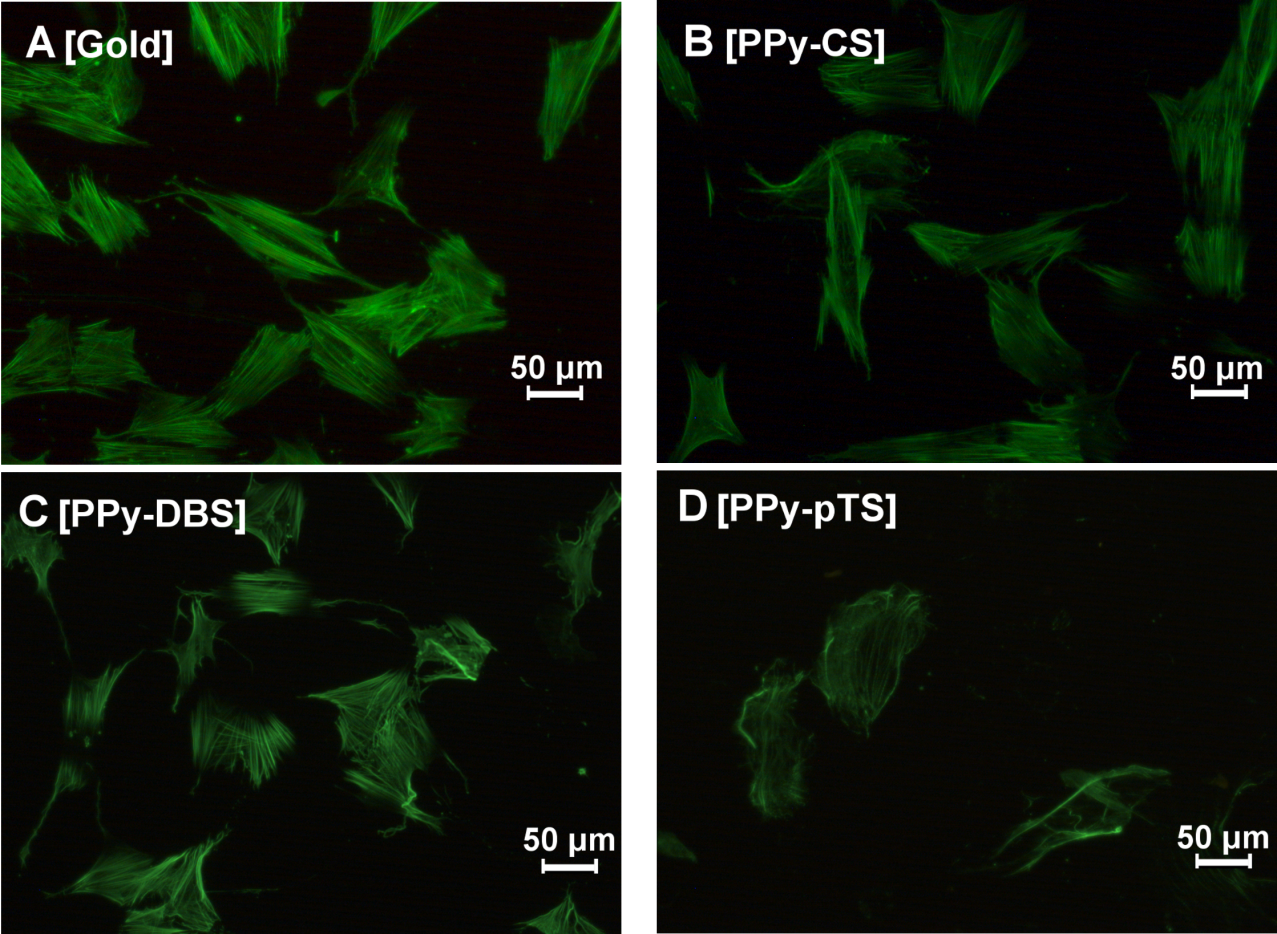
Osteoblast morphology staining. Representative F-actin filament staining with Alexa488 phalloidin [A-D] of gold and Polypyrrole (PPy) doped with chondroitin sulfate (CS), dodecylbenzenesulfonate (DBS) and p-Toluene sulfonate (pTS). The magnification is 20-fold.

The morphology of adherent osteoblasts on the different material surfaces was visualized by fluorescent staining of cytoskeletal F-actin filaments after 72 h 1α,25-(OH)_2_D_3_ treatment to induce an osteogenic phenotype. F-actin morphology illustrated that gold, PPy-DBS, and PPy-CS were highly potent in maintaining an osteoblastic phenotype (Fig 3A–3C). This finding was strengthened by the well-visible F-actin filament network and the developed filopodia-fiber extensions. In contrast, osteoblasts grown on PPy-pTS (Fig 3D) were enlarged with disorganized actin filaments. This suggested that pTS prevents the formation of an appropriate environment for osteoblast attachment. The increased size as a result of cell spreading might indicate that the osteoblasts are desperately trying to find spots to attach [32]. An inappropriate growing potential was further demonstrated by the decreased number of osteoblasts attached on PPy-CS or PPy-pTS.

**Fig 4:**
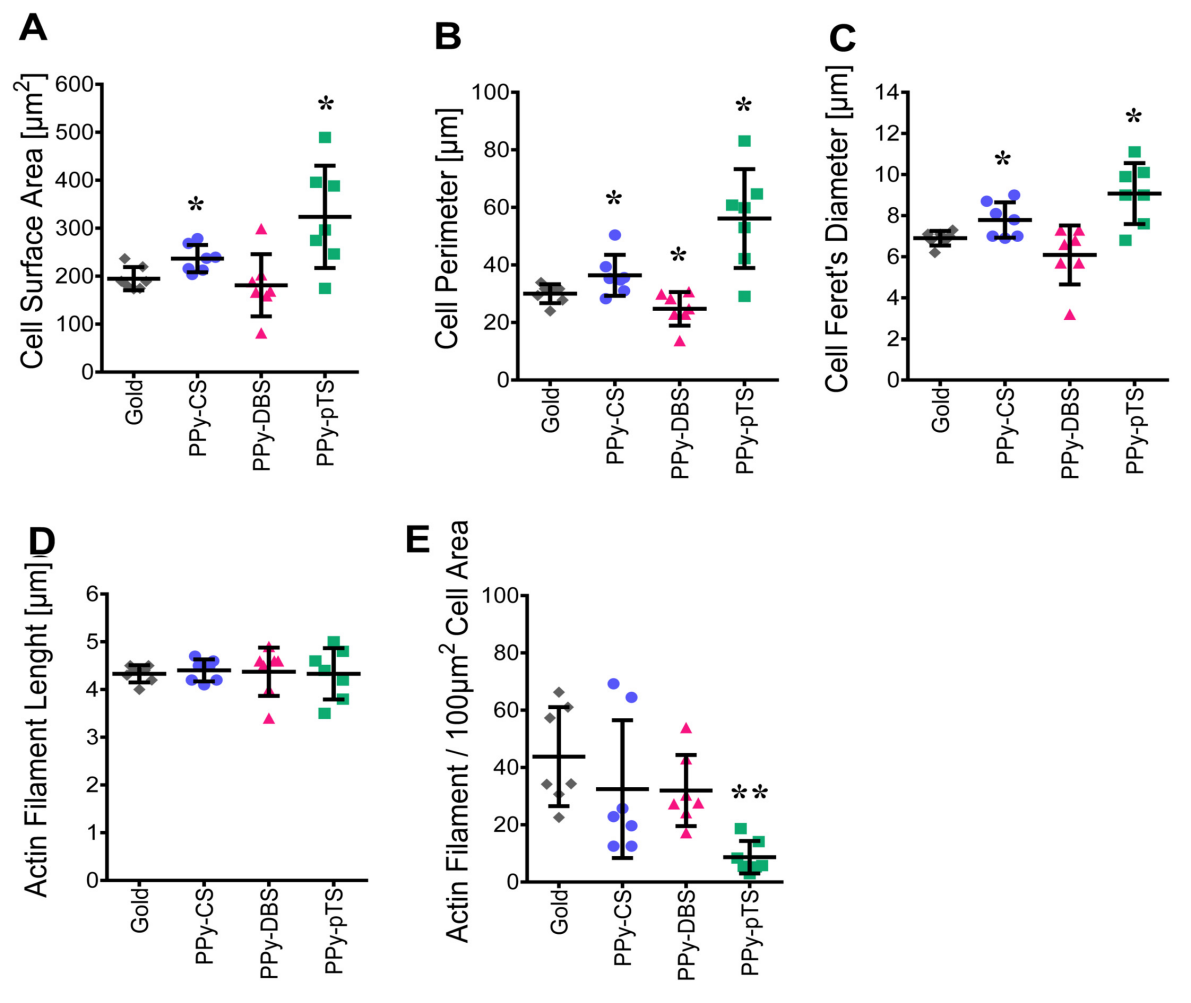
Cell morphology analysis. Area [A], Perimeter [B] and Feret’s diameter [C] was analyzed with Fiji ImageJ. F-actin filament quantification in terms of length [D] and number/10μm^2^ cell area [E] with FilaQuant. The value represents mean ± standard deviation (n = 5). *p<0.05 v. gold control.

The actin cytoskeletal properties of osteoblasts adhered to different material surfaces were analyzed by measuring different parameters of fluorescent F-actin. The F-actin area, perimeter, filament length, number of F-actin filaments/100 μm^2^ cell area, and Feret’s diameter of the cells on PPy-DBS were similar as on the gold-coated surface (Fig 4A–4E). On the other hand, PPy-pTS and PPy-CS resulted in increased F-actin area, perimeter, and Feret’s diameter, suggesting that the cells were enlarged and spreading. Although the F-actin was disorganized, the cells kept their osteogenic function on these dopants, suggesting that they were still tolerated by human primary osteoblasts (Fig 3A and 3C). Elongation of F-actin filaments occurs in cells that are stably anchored to their substrate. This mechanism provides an enhanced connection to focal adhesions that further strengthen cell attachment [32]. In the current study there was no difference in the length or total area of the F-actin filaments. However, beside the length of the actin filament for osteoblast attachment and migration, the physically connection to the surface by connector proteins are crucial for focal adhesion.

**Fig 5:**
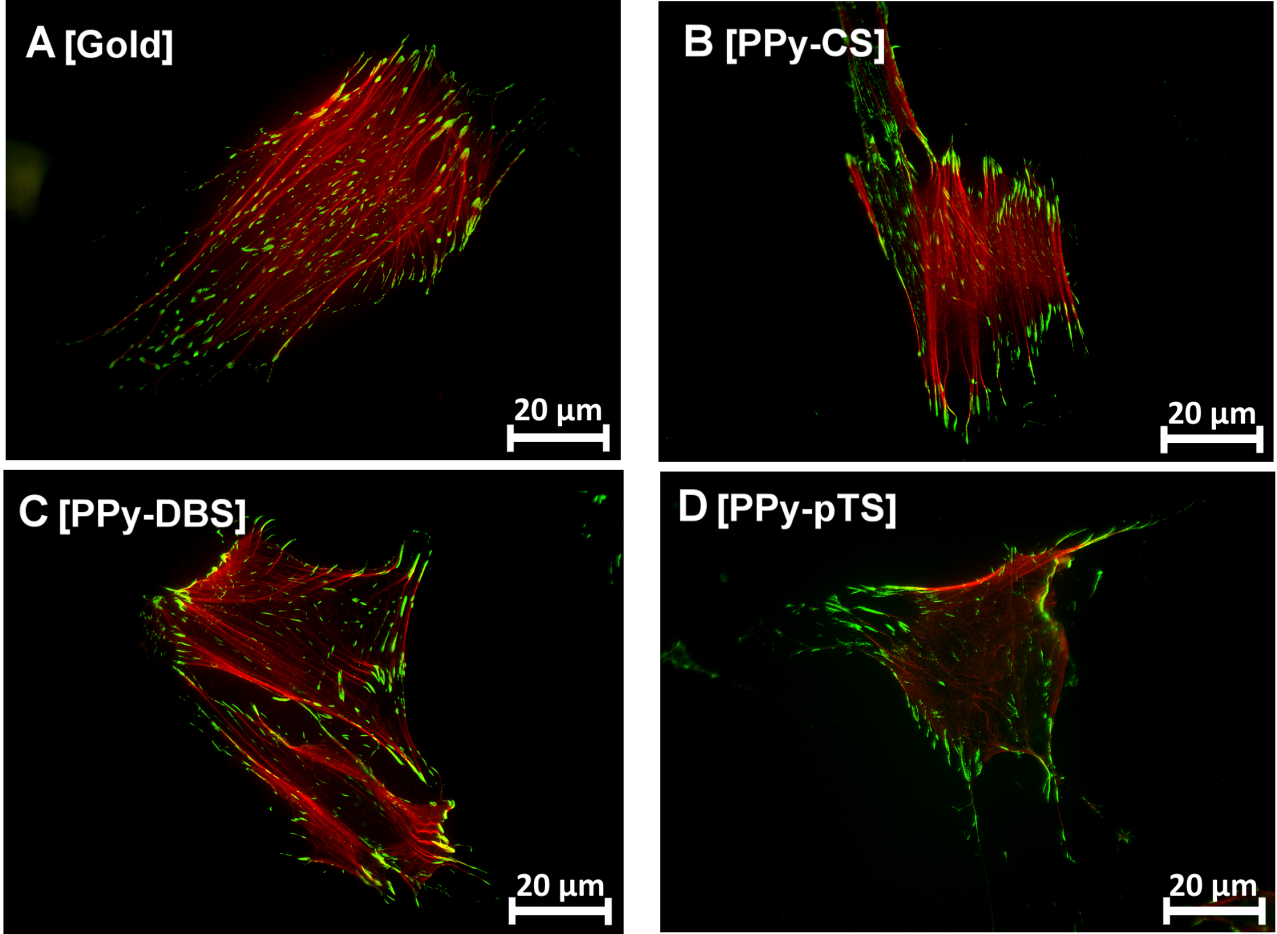
Vinculin staining. Representative vinculin staining of attachment points, visualized with Alexa Flour 488(green) and F-actin filament staining with Alexa Fluor 568 phalloidin(red) of gold [A] and Polypyrrole (PPy) doped with chondroitin sulfate (CS) [B], dodecylbenzenesulfonate (DBS) [C] and p-Toluene sulfonate (pTS) [D]. The magnification is 63-fold.

**Fig 6:**
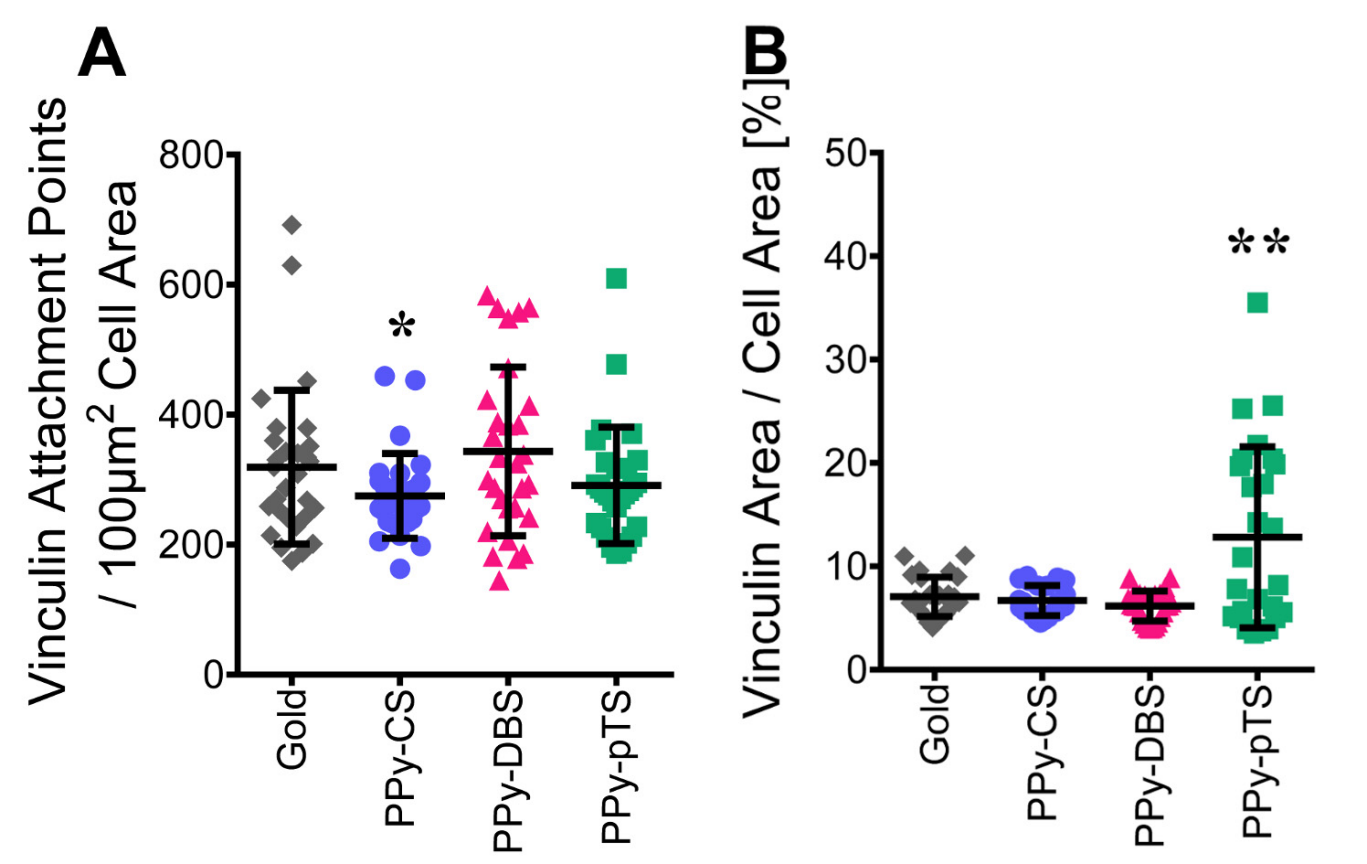
Focal adhesion and attachment. Vinculin attachment points / 100 μm^2^ cell area [A] and percentage share of vinculin area / cell area [B] was analyzed with Fiji ImageJ. The value represents mean ± standard deviation (n = 27). *p<0.05, **p<0.01 v. gold control.

F-actin filaments and stress fibers are connected by focal adhesions via integrins to the biomaterial surface. Focal adhesions are organized protein complexes that physically connect the extracellular matrix to the cytoskeleton. This connection is crucial for osteoblasts to maintain their morphology and osteogenic potential. To better understand the distribution of focal adhesions in osteoblasts, the focal adhesion protein vinculin was labeled with fluorescent antibodies. Vinculin couples, transmits, transduces, and regulates mechanical forces between the cytoskeleton and adhesion receptors. The size of the vinculin complex has been suggested to predict cell migration [33]. Microscopic analysis of our samples showed a punctuated arrangement of labeled vinculin attachment points linked to F-actin, which is typical for focal adhesion complexes (Fig 5A–5D). Osteoblasts seeded on PPy-DBS or PPy-pTS showed the same number of labeled vinculin attachment points per cell area as gold (Fig 6A), while cells on PPy-CS showed a reduced number of vinculin attachment points per cell area as gold. The osteoblasts on PPy-pTS had a larger labeled vinculin area per cell area (Fig 6B) than osteoblasts on the other material surfaces tested. Large spots of vinculin in a non-appropriate environment might be considered as a stress response by adhering cells to withstand mass cellular detachment from an implant surface [34]. Indeed, detachment of osteoblasts from PPy-pTS was demonstrated by changed morphological features and decreased DNA content.

Materials that stimulate osteoblast attachment are promising for enhanced osseointegration. Bone implants coated with bisphosphates show stronger fixation and more surrounding bone in humans, which might lead to new strategies for orthopaedic surgery and dental implant placement in osteoporotic bone [35]. Moreover, local delivery of zoledronic acid has been used as antitumor treatment [36]. Local delivery of bisphosphonates into areas with high bone degradation, such as bone tumors or peri-prosthetic osteolytic zones, opens up the possibility for treatment using higher dosages locally while avoiding systemic side effects. Interestingly, the current PPy-material could be charged with bisphosphonates that would be released upon electric stimulation.

### Osteogenic function

The field of dentistry is a highly promising area of application for conducting polymers. The primary stability and surface microtopography of dental implants are crucial to induce early bone healing with osteoblasts attached to the implant surface. Efficient osteoblast adhesion and attachment to the bone implant is important since it induces a fast healing response. During differentiation and maturation of osteoblasts, the transcription factor RUNX2 is expressed. A mature osteoblast functions by producing ECM containing the glycoproteins SPARC and OPN, COL1A, OCN and the enzyme ALP, which is involved in ECM calcification. Enhancement of osteoblast activity with controlled drug release from an implant coated with conducting polymers can open up strategies to enhance osseointegration and implant stability in patients with bad or low bone stock.

**Fig 7:**
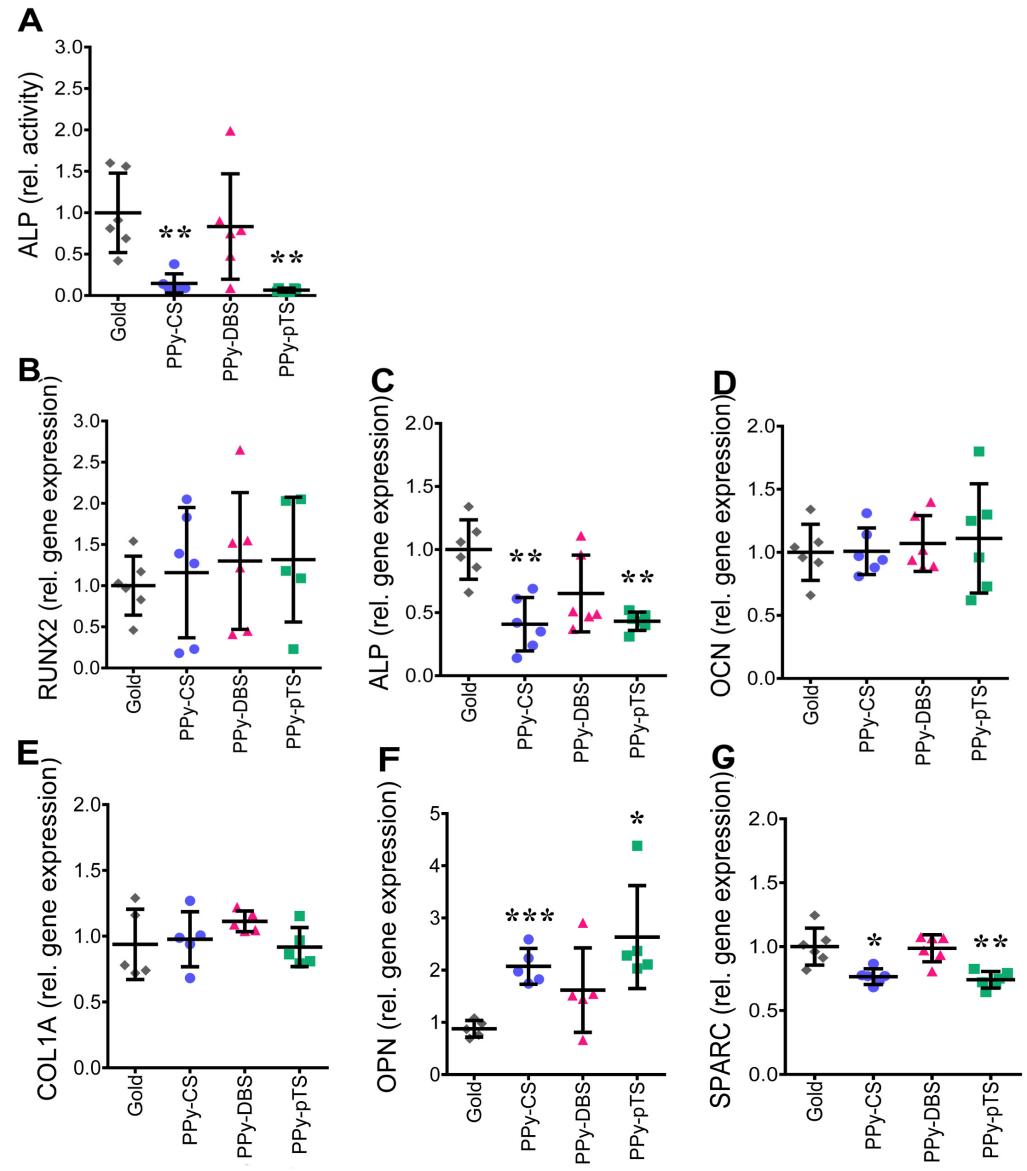
Osteogenic potential. ALP activity measurement [A] of osteoblasts grown on gold and Polypyrrole (PPy) doped with chondroitin sulfate (CS), dodecylbenzenesulfonate (DBS) and p-Toluene sulfonate (pTS). Gene expression of the bone specific genes RUNX2 [B], ALP [C], OCN [D], COL1A [E], OPN [F] and SPARC [G]. Gene expression levels were normalized based on the square root of the product formed by HPRT1 and YWHAZ and represents mean ± standard deviation normalized to gold control (n≥4). *p<0.05, **p<0.01 and ***p<0.001 v. gold control.

Osteoblast function was analyzed by measuring gene expression of the transcription factor RUNX2, the glycoproteins OCN and OPN, the gene coding COL1A and the differentiation markers ALP and OCN. ALP is the basic phosphatase in bone and is crucial for osteoblast function. Gene expression and ALP enzyme activity of osteoblasts seeded on PPy-DBS was similar to that of osteoblasts seeded on a gold surface, suggesting that this dopant was maintaining the osteogenic potential (Fig 7A and 7C). In contrast, PPy-pTS and PPy-CS suppressed ALP gene and protein expression compared to gold. Moreover, ALP activity of osteoblasts grown on PPy-CS and PPy-pTS was below the detection limit in 2 out of 3 donors, suggesting that these materials were not favorable for osteoblasts.

COL1A is the most dominant component in the bone matrix, providing a favorable ECM for progenitor cells [37], but RUNX2 is the most important transcription factor to induce osteogenesis by activating downstream differentiation marker genes via multiple interactions [38]. RUNX2 and COL1A gene expression was similar for all dopants compared to the gold surface (Fig 7B and 7E), suggesting that all materials tested were tolerated by the osteoblasts and prevented de-differentiation.

SPARC supports osteoblast survival, is able to strengthen osteoblastic lineage commitment, and modulates the balance of bone formation and resorption. SPARC expression decreases after termination of ECM mineralization [39]. OPN is a glycoprotein, which is maximally expressed during the mineralization process. It is known to enhance osteoclastogenesis and to inhibit mineralization. SPARC and OPN expression was similar in osteoblasts seeded on PPy-DBSA and on a gold surface. SPARC expression deceased in osteoblasts seeded on PPy-CS and PPy-pTS compared to gold (Fig 7G), while OPN gene expression increased in osteoblasts grown on PPy-CS and PPy-pTS compared to gold (Fig 7F). OCN is a late stage marker of osteoblast differentiation and is associated with enhanced mineralization due to its ability to bind calcium and hydroxyapatite. There is evidence that OCN is able to compensate a lack of ALP activity during the mineralization process [37]. OCN enhances osteochondrogenic differentiation and thereby calcification in the presence of vitamin D [40]. Cells on all dopants expressed similar amounts of OCN compared to cells on the gold surface (Fig 7D). Cells on both PPy-pTS and PPy-CS showed an up-regulation of OPN expression and a downregulation of SPARC, while OCN gene expression was unaffected. The differential expression of these matrix proteins could be explained by the epigenetic state of the osteogenic genes. Silencing of certain histone deacetylases (HDAC) by valproic acid, has been shown to directly promote the availability of osteogenetic markers such as OPN and OCN [41]. Mechanical stimulation can induce epigenetic changes and promote availability of osteogenetic genes [42]. In current study, the dopants might affect HDAC, thereby altering DNA methylation and the expression of factors such as OPN and OCN. Using dopants on implants or bone grafts, which allow a controlled release of drugs by electric stimulation might be an option for direct manipulation of the epigenetic state of cells.

In conclusion, all dopants tested showed similar gene expression levels of the early transcription factor RUNX2, the bone matrix protein OCN, and COL1A, compared to a gold surface (Fig 7B, 7D and 7E), suggesting that all materials were tolerated by the osteoblasts. Only PPy-DBS, but not the other dopants tested, showed similar gene expression of OPN, SPARC, and ALP compared to gold. PPy-pTS and PPy-CS also revealed increased OPN gene expression levels,that might indicate less favorable osteogenic properties. All together these data suggests that PPy-DBS might be more promising for osseointegration than PPy-pTS and PPy-CS.

## Conclusion

Local delivery of pharmaceutics can reach the bone cells or the infection area around the implant more efficiently than systemic delivery. Local delivery of bisphosphonates has shown remarkable results in orthodontic implants [35], which might open up treatment alternatives for patients that are not allowed to receive specific systemic doses of pharmaceuticals due to side effects. Moreover, improved cell adhesion and differentiation is crucial to improve implant osseointegration. The ultimate goal of the current study was to find a material that is biocompatible with human osteoblasts, and that has the potential to be used as a drug delivery system. Besides local delivery of drugs released from the implant, polypyrrole could be coated on electro-spun nanofibers for scaffolds. Scaffolds could be seeded with either osteoblasts or stem cells in the presence or absence of different drugs for bone regeneration [43]. Dental pulp stem cells have been suggested as a promising source of stem cells and have recently been used to restore bone defects in the oralfacial region [44]. We showed that all dopants tested were tolerated by human primary osteoblasts. However, PPy incorporated with the dopant DBS provides the most potent material surface for focal adhesion, and maintains the osteoblast-like phenotype of human primary osteoblasts. PPy-CS and PPy-pTS showed less ability to maintain the osteoblast phenotype. This demonstrates that PPy-coated implants doped with DBS have great potential for the (re)generation of bone tissue by providing electrical stimulation and/or controlled release of drugs or growth factors. The cells used in this study were harvested from patients who underwent primary hip arthroplasty, thereby reflecting a representative bone stock of patients with need for both dental and orthopedic applications. Therefore, conducting polymers might provide a novel and promising material for the design of orthopedic or dental implants that can fight infections, or focally enhance bone formation in a tightly controlled manner. The next step in our investigation will be to study the effect of a controlled release of antibiotics and/or growth factors from DBS-doped PPy materials on primary osteoblasts.

## Supporting Information

S1 File Optimization of Images prior FilaQuant analysis. A detailed description of performed optimizations of the images prior the semi-quantitative analysis by FilaQuant is provided which ensured an improved detection of the stained F-actin filaments in the obtained images.
(DOCX)

S2 File Image processing for vinculin analysis by Fiji ImageJ. A detailed description of performed optimizations of the images prior the semi-quantitative analysis by Fiji ImageJ is provided which ensured an improved detection of the stained vinculin attachment points in the obtained images.
(DOCX)

S1 Table FilaQuant Settings. An overview of the FilaQuant preferences for the semi-quantitative analysis of F-actin filaments is given.
(DOCX)

S1 Dataset Experimental Data. The experimental data which led to the findings in the paper are provided.
(XLSX)

## References

[1] Mombelli A, Müller N, Cionca N. The epidemiology of peri-implantitis. Clin Oral Implants Res. 2012;23 Suppl 6:67–76. 10.1111/j.1600-0501.2012.02541.x 23062130

[2] Ateh DD, Navsaria HA, Vadgama P. Polypyrrole-based conducting polymers and interactions with biological tissues. J R Soc Interface. 2006;3:741–52. 17015302 10.1098/rsif.2006.0141 PMC1885362

[3] Gelmi A, Higgins MJ, Wallace GG. Physical surface and electromechanical properties of doped polypyrrole biomaterials. Biomaterials. 2010;31:1974–83. 10.1016/j.biomaterials.2009.11.040 20056273

[4] Tourillon G, Garnier F. Effect of Dopant on the Physicochemical and Electrical Properties of Organic Conducting Polymers. J Phys Chem. 1983;87:2289–92.

[5] Ghosh P, Han G, De M, Kim CK, Rotello VM. Gold nanoparticles in delivery applications. Adv Drug Deliv Rev. 2008;60:1307–15. 10.1016/j.addr.2008.03.016 18555555

[6] Zhang J, Neoh KG, Hu X, Kang E-T, Wang W. Combined effects of direct current stimulation and immobilized BMP-2 for enhancement of osteogenesis. Biotechnol Bioeng. 2013;110:1466–75. 10.1002/bit.24796 23192383

[7] Gelmi A, Ljunggren MK, Rafat M, Jager EWH. Influence of conductive polymer doping on the viability of cardiac progenitor cells. J Mater Chem B. 2014;2:3860–67. 10.1039/c4tb00142g 32261732

[8] Svennersten K, Berggren M, Richter-Dahlfors A, Jager EWH. Mechanical stimulation of epithelial cells using polypyrrole microactuators. Lab Chip. 2011;11:3287–93. 10.1039/c1lc20436j 21842071

[9] Guimard NK, Gomez N, Schmidt CE. Conducting polymers in biomedical engineering. Prog Polym Sci. 2007;32:876–921.

[10] Heinze J. Electrochemistry of conducting polymers. J Synth Met. 1991;43:2805–23.

[11] Tsai S-W, Chen C-C, Liou H-M, Hsu F-Y. Preparation and characterization of microspheres comprised of collagen, chondroitin sulfate, and apatite as carriers for the osteoblast-like cell MG63. J Biomed Mater Res A. 2010;93:115–22. 10.1002/jbm.a.32502 19536833

[12] Pecchi E, Priam S, Mladenovic Z, Gosset M, Saurel a-S, Aguilar L, et al A potential role of chondroitin sulfate on bone in osteoarthritis: inhibition of prostaglandin E_2_ and matrix metalloproteinases synthesis in interleukin-1β-stimulated osteoblasts. Osteoarthritis Cartilage. 2012;20:127–35. 10.1016/j.joca.2011.12.002 22179028

[13] Fonner JM, Forciniti L, Nguyen H, Byrne JD, Kou Y-F, Syeda-Nawaz J, et al Biocompatibility implications of polypyrrole synthesis techniques. Biomed Mater. 2008;3:034124 10.1088/1748-6041/3/3/034124 18765899 PMC2562301

[14] Han D-H, Lee HJ, Park S-M. Electrochemistry of conductive polymers XXXV: Electrical and morphological characteristics of polypyrrole films prepared in aqueous media studied by current sensing atomic force microscopy. Electrochim Acta. 2005;50:3085–92.

[15] Warren LF, Anderson DP. Polypyrrole Films from Aqueous Electrolytes The Effect of Anions upon Order. J Electrochem Soc. 1987;134:101–5.

[16] Santos A, Bakker AD, Willems HME, Bravenboer N, Bronckers ALJJ, Klein-Nulend J. Mechanical loading stimulates BMP7, but not BMP2, production by osteocytes. Calcif Tissue Int. 2011;89:318–26. 10.1007/s00223-011-9521-1 21842277

[17] Schindelin J, Arganda-Carreras I, Frise E, Kaynig V, Longair M, Pietzsch T, et al Fiji: an open-source platform for biological-image analysis. Nat Methods. 2012;9:676–82. 10.1038/nmeth.2019 22743772 PMC3855844

[18] Zack GW, Rogers WE, Latt SA. Automatic measurement of sister chromatid exchange frequency. J Histochem Cytochem. 1977;25:741–53. 70454 10.1177/25.7.70454

[19] Matschegewski C, Staehlke S, Birkholz H, Lange R, Beck U, Engel K, et al Automatic Actin Filament Quantification of Osteoblasts and Their Morphometric Analysis on Microtextured Silicon-Titanium Arrays. Materials. 2012;5:1176–95.

[20] Hennig S, Duffull SB. Different ways of incorporating data below the quantification limit in datasets for parameter estimation. PAGANZ 07: Population Approach Group in Australia and New Zealand. Singapore; 2007.

[21] Balint R, Cassidy NJ, Cartmell SH. Conductive polymers: Towards a smart biomaterial for tissue engineering. Acta Biomater. 2014;10:2341–53. 10.1016/j.actbio.2014.02.015 24556448

[22] Thompson BC, Moulton SE, Richardson RT, Wallace GG. Effect of the dopant anion in polypyrrole on nerve growth and release of a neurotrophic protein. Biomaterials. 2011;32:3822–31. 10.1016/j.biomaterials.2011.01.053 21353699

[23] Vandrovcová M, Bačáková L. Adhesion, growth and differentiation of osteoblasts on surface-modified materials developed for bone implants. Physiol Res. 2011;60:403–17. 21401307 10.33549/physiolres.932045

[24] Hempel U, Hefti T, Dieter P, Schlottig F. Response of human bone marrow stromal cells, MG-63, and SaOS-2 to titanium-based dental implant surfaces with different topography and surface energy. Clin Oral Implants Res. 2013;24:174–82. 10.1111/j.1600-0501.2011.02328.x 22092368

[25] Costa DO, Prowse PDH, Chrones T, Sims SM, Hamilton DW, Rizkalla AS, et al The differential regulation of osteoblast and osteoclast activity by surface topography of hydroxyapatite coatings. Biomaterials. 2013;34:7215–26. 10.1016/j.biomaterials.2013.06.014 23830579

[26] Chang E-J, Kim H-H, Huh J-E, Kim I-A, Seung Ko J, Chung C-P, et al Low proliferation and high apoptosis of osteoblastic cells on hydrophobic surface are associated with defective Ras signaling. Exp Cell Res. 2005;303:197–206. 15572039 10.1016/j.yexcr.2004.09.024

[27] Davies JE. Understanding peri-implant endosseous healing. J Dent Educ. 2003;67:932–49. 12959168

[28] Zhu X, Chen J, Scheideler L, Reichl R, Geis-Gerstorfer J. Effects of topography and composition of titanium surface oxides on osteoblast responses. Biomaterials. 2004;25:4087–103. 15046900 10.1016/j.biomaterials.2003.11.011

[29] Stoddart MJ. Mammalian Cell Viability: Methods and Protocols Methods in Molecular Biology. Humana Press,; 2011.

[30] Sirivisoot S, Pareta R, Webster TJ. Electrically controlled drug release from nanostructured polypyrrole coated on titanium. Nanotechnology. 2011;22:085101 10.1088/0957-4484/22/8/085101 21242621

[31] Liu WF. Mechanical regulation of cellular phenotype: implications for vascular tissue regeneration. Cardiovasc Res. 2012;95:215–22. 10.1093/cvr/cvs168 22628449

[32] Hill DA, Chiosea S, Jamaluddin S, Roy K, Fischer AH, Boyd DD, et al Inducible changes in cell size and attachment area due to expression of a mutant SWI/SNF chromatin remodeling enzyme. J Cell Sci. 2004;117:5847–54. 15537831 10.1242/jcs.01502

[33] Kim DH, Wirtz D. Focal adhesion size uniquely predicts cell migration. FASEB J. 2013;27:1351–1361. 10.1096/fj.12-220160 23254340 PMC3606534

[34] Yue C, van der Mei HC, Kuijer R, Busscher HJ, Rochford ETJ. Mechanism of cell integration on biomaterial implant surfaces in the presence of bacterial contamination. J Biomed Mater Res Part A. 2015;0:1–9. 10.1002/jbm.a.35502 25966819

[35] Abtahi J, Tengvall P, Aspenberg P. A bisphosphonate-coating improves the fixation of metal implants in human bone. A randomized trial of dental implants. Bone. 2012;50:1148–51. 10.1016/j.bone.2012.02.001 22348981

[36] Marra M, Salzano G, Leonetti C, Porru M, Franco R, Zappavigna S, et al New self-assembly nanoparticles and stealth liposomes for the delivery of zoledronic acid: A comparative study. Biotechnol Adv. 2012;30:302–9. 10.1016/j.biotechadv.2011.06.018 21741464

[37] Arpornmaeklong P, Brown SE, Wang Z, Krebsbach PH. Phenotypic characterization, osteoblastic differentiation, and bone regeneration capacity of human embryonic stem cell-derived mesenchymal stem cells. Stem Cells Dev. 2009;18:955–68. 10.1089/scd.2008.0310 19327009 PMC3032563

[38] Kalajzic I, Staal A, Yang WP, Wu Y, Johnson SE, Feyen JHM, et al Expression profile of osteoblast lineage at defined stages of differentiation. J Biol Chem. 2005;280:24618–26. 15834136 10.1074/jbc.M413834200

[39] Hojo H, Ohba S, Chung U. Signaling pathways regulating the specification and differentiation of the osteoblast lineage. Regen Ther. 2015;1:57–62. 10.1016/j.reth.2014.10.002 PMC6581763 31245441

[40] Kapustin AN, Shanahan CM. Osteocalcin: a novel vascular metabolic and osteoinductive factor? Arterioscler Thromb Vasc Biol. 2011;31:2169–71. 10.1161/ATVBAHA.111.233601 21918209

[41] Paino F, La Noce A, Tirino I, Naddeo A, Desiderio V, Pirozzi U, et al Histone deacetylase inhibition with valproic acid downregulates osteocalcin gene expression in human dental pulp stem cells and osteoblasts: Evidence for HDAC2 involvement. Stem Cells. 2014;32:279–89. 10.1002/stem.1544 24105979 PMC3963447

[42] Chen JC, Chua M, Bellon RB, Jacobs CR. Epigenetic Changes During Mechanically Induced Osteogenic Lineage Commitment. J Biomech Eng. 2015;137:020902 10.1115/1.4029551 25581684 PMC4321109

[43] Gelmi A, Zhang J, Cieslar-Pobuda A, Ljunngren MK, Los MJ, Rafat M, et al Electroactive polymer scaffolds for cardiac tissue engineering. Electroactive Polymer Actuators and Devices (EAPAD). 2015;9430:94301T-1.

[44] La Noce M, Mele L, Tirino V, De Rosa A, Naddeo P, Papagerakis P, et al Neural Crest Stem Cell Population In Craniomaxillofacial Development And Tissue Repair. Eur Cells Mater. 2014;28:348–57. 10.22203/ecm.v028a24 25350250

