# Supplementary material for: Biocompatibility of Polypyrrole with Human Primary Osteoblasts and the Effect of Dopants
Source: PLoS One. 2015 Jul 30;10(7):e0134023. doi: 10.1371/journal.pone.0134023 (PMC4520445; doi:10.1371/journal.pone.0134023)
Supplement: S1 File — A detailed description of performed optimizations of the images prior the semi-quantitative analysis by FilaQuant is provided which ensured an improved detection of the stained F-actin filaments in the obtained images. (DOCX) [file pone.0134023.s001.docx]

**Image processing for F-Actin filament analysis by Fiji Image J for FilaQuant analysis**

After loading the Image into Fiji Image J, the background was subtracted, using a rolling ball radius of 30 pixels. Secondly, the image calculation function was used to add the identical picture followed by enhancing the contrast using a pixel saturation of 0.05%. Thirdly, the color channels got split, and the result of the green channel was used for further analysis by FilaQuant after an additional contrast-enhancing step with a pixel saturation of 0.7%. In FilaQuant the Images were preprocessed by Top-Hat Transformation followed by using the Ridge Detector Method.
