# Supplementary material for: Biocompatibility of Polypyrrole with Human Primary Osteoblasts and the Effect of Dopants
Source: PLoS One. 2015 Jul 30;10(7):e0134023. doi: 10.1371/journal.pone.0134023 (PMC4520445; doi:10.1371/journal.pone.0134023)
Supplement: S2 File — A detailed description of performed optimizations of the images prior the semi-quantitative analysis by Fiji ImageJ is provided which ensured an improved detection of the stained vinculin attachment points in the obtained images. (DOCX) [file pone.0134023.s002.docx]

**Image processing for Vinculin analysis by Fiji Image J**

After loading the Image into Fiji Image J, the background was subtracted, using a rolling ball radius of 25 pixels. Secondly, the contrast was enhancing using a pixel saturation of 0.4%. After converting the image into 8- bit, the Auto Threshold Triangle Method was used, images binarized and subsequent inverted. The analysis of the Vinculin was performed by using the analyze particle feature applying the outline setting. Results were summarized for each cell and used for further analysis.
