# Supplementary material for: Biocompatibility of Polypyrrole with Human Primary Osteoblasts and the Effect of Dopants
Source: PLoS One. 2015 Jul 30;10(7):e0134023. doi: 10.1371/journal.pone.0134023 (PMC4520445; doi:10.1371/journal.pone.0134023)
Supplement: S1 Table — An overview of the FilaQuant preferences for the semi-quantitative analysis of F-actin filaments is given. (DOCX) [file pone.0134023.s003.docx]

**FilaQuant® preferences for semi-quantitative analysis of F-actin filaments**

| Top-Hat Radius: | 5 |
| --- | --- |
| Noise Penalizer: | 0.2 |
| Optimizer Iterations: | 200 |
| Max. Vertex Laplacian: | -50 |
| Min. Ridgeness: | 50 |
| Min. Edge-Length: | 5 |
| Max. Edge-Length: | 60 |
| Filament Model Width: | 1.5 |
| Checkerboard Penalizer: | 0.1 |
| Intensity Threshold: | 30 |
| Random Filament Number: | 0 |
| Gaussian Filter Size: | 1 |
| 1 Per Pixel: | 1 |
